# Progress in Gynæcology

**Published:** 1901-09-14

**Authors:** 


### PROGRESS IN GYNÆCOLOGY.

**The Ovary as an Organ of Internal Secretion.**—It is now generally admitted that the ovaries exert a decided influence over the organism as a whole, and all evidence is strongly in favour of the view that this influence is the result of an internal secretion which favours katabolic changes. Castration, therefore, both in the male and female, deprives the organism of a stimulant of oxydation, but no chemical substance has been isolated to which the properties of such an internal secretion can be ascribed. W. E. Dixon[1] says that from a study of clinical evidence three points seem to be clearly demonstrated: First, that the presence in the body of ovarian tissue, however small in amount, is sufficient to prevent the distressing symptoms which frequently arise after complete double ovariotomy; secondly, the administration of ovarian tissue by the mouth exerts a beneficial effect in patients in whom menstruation has ceased in consequence of disease or complete ovariotomy; thirdly, ovariotomy has a distinct effect on metabolism. Roubinstein,[2] from a series of experiments in rabbits and dogs, found that in the majority of cases removal of one ovary was followed by marked compensatory hypertrophy of the remaining one. Microscopically, a decided increase in the number of Graafian follicles and corpora lutea were observed. No anatomical changes in the uterus resulted from extirpation of one ovary. S. W. Bandler[3] maintains that ovarian secretion exerts a most decided effect upon the development of the uterus and the genitalia, as well as upon the breasts. It is, he says, absolutely necessary for the preservation of the developed uterus, the other genitalia, and the breasts. An over-production of ovarian secretion or a disturbance in the function of the ovary causes pathological changes; an under-production of ovarian secretion is likewise the cause of pathological conditions. He quotes an experiment by Ribbert who implanted the mamma of a young guinea-pig with its covering of skin into a cut near the ear. The wound healed, and five months after the operation, the animal having borne two young, this mamma secreted milk normally—a proof that the connection between the breasts and the ovary and uterus is to be found in no other channels than those of the circulation.

**Prolapse of the Uterus.**—T. Wilson[4] emphasises the fact that the so-called prolapse of the uterus is really a hernia, brought about by the same exciting causes as other herniæ, and due to the yielding of a particular part of the abdominal wall, viz., the pelvic floor. With regard to treatment, after drawing attention to the great importance of preventing this complication by the correct management of the patient during pregnancy and labour, and for two or three months afterwards, he says that when prolapse is present treatment should be undertaken at the earliest possible moment. He condemns the use of vaginal pessaries of all kinds as being liable to set up vaginitis, and exert an injurious influence on the nutrition of the surrounding tissues. In early cases he recommends the use of glycerine tampons, which the patient is instructed how to make and introduce for herself every morning. This method, he says, besides affording the necessary support, has two conspicuous advantages over the pessary treatment; in the first place it is cleanly, and in the second it exerts on the tissues a bracing and tonic action which helps to prevent the further progress of the prolapse. When this fails he would support the pelvic floor on the outside, by a perineal pad, just as one supports any other hernia by a truss. When prolapse has become firmly established, and these methods are ineffectual, he advocates operative treatment; those operative procedures most useful being anterior colporrhaphy, posterior colporrhaphy, perineorrhaphy, amputation of the cervix, and anterior fixation of the fundus of the uterus to the vesical peritoneum, according to the variety of prolapse present. H. J. Boldt[5] says that he considers ventral fixation the most satisfactory operation for prolapse, the operation being done in such a way as to give a broad and firm adhesion between the abdominal parieties and the uterus. As a rule a plastic operation on the vagina is not required after ventral fixation. Axel Wallgren[6] recommends in patients before the climacteric ventral fixation and resection of the vagina, at or after the menopause, either ventral fixation or hysterectomy may be favourably practised, but if any weakening of the abdominal wall is present it will be found best to perform hysterectomy and then resect the vagina.

**Histogenesis of Ovarian Dermoids.**—W. D. Haggard[7] believes that ovarian dermoids have a different origin to those met with in other parts of the body—*e.g.*, the orbit, pharynx, scrotum, testis, etc.—for whereas these probably result from the inclusion of the ectoderm in the development of the embryo, and contain purely dermal structures, those of the ovary contain derivatives of both the mesoderm and enteroderm, and are more akin to teratomata. He thinks that some pathological activity on the part of the ovum in the Graafian follicle is responsible.

[1] Practitioner, May, 1901. [2] Am. Jour. of Med. Sc., March, 1901. [3] Med. Rec., March 16, 1901. [4] Bir. Med. Rev., March, 1901. [5] Med. Rec., Jan., 1901. [6] Brit. Med. Jour., Feb. 16, 1901. [7] Edin. Med. Jour., April, 1901.

